# The impact of energy releasing B-vitamin intake on indices of obesity and cardiac function: a cross-sectional study

**DOI:** 10.12688/f1000research.139672.3

**Published:** 2024-08-06

**Authors:** Lana M. Agraib, Islam Al-Shami, Buthaina Alkhatib, Hanan Hasan

**Affiliations:** 1 Department of Food Technology and Nutrition, Faculty of Agriculture, Jerash University, Jerash, Jerash Governorate, Jordan; 2 Department of Clinical Nutrition and Dietetics, Faculty of Applied Medical Sciences, The Hashemite University, Zarqa, Jordan; 3 The University of Jordan, Amman, Jordan

**Keywords:** energy-releasing vitamins, obesity indices, cardiac function indices, abdominal volume index, body roundness index

## Abstract

**Background:**

B vitamins play a crucial role in the balance and metabolism of energy. Energy metabolism mainly benefits from the B-complex vitamins. Specifically, decarboxylation, transamination, acylation, oxidation, and reduction of substrates that are ultimately employed in energy intake require thiamin, riboflavin, niacin, and vitamin B6. Vitamin deficiency could lead to chronic disease occurrence.

**Objectives:**

To assess the impact of energy-releasing B-vitamins intake (B1, B2, B3, and B6) on selected indices of obesity and cardiac function.

**Methods:**

A cross-sectional study was performed on 491 apparently healthy adults (18-64 years old) between January and May 2019 at Hashemite University, Jordan. Anthropometric measurements were taken, lipid profiles were analyzed, and indices of obesity and cardiac function were calculated. The typical dietary intake of B1, B2, B3, and B6 vitamins was calculated.

**Results:**

Conicity index (CI) and abdominal volume index (AVI) scores significantly decreased with the increased adjusted vitamin B1 and B6 intake. Also, body roundness index (BRI), weight-adjusted-waist index (WWI), lipid accumulation product (LAP), and atherogenic index of plasma (AIP) scores were decreased with the increase of adjusted B6 intake ( *p*<0.05). The total sample showed a significant inverse weak correlation between energy-adjusted intake of B1 and AVI (r= -0.156, *p*=0.001) and BRI (r= 0.111, *p*=0.014). Similar correlations were detected among male participants between energy-adjusted B1 intake and BAI, AVI, and BRI. Female participants had a significant weak inverse correlation between BAI and energy-adjusted B2 (r= -0.180, *p*=0.029) and B6 intake (r= -0.212, *p*=0.010). Only B1, the vitamin, significantly explained 2.43 and 1.24% of changes observed in the AVI and BRI scores, respectively ( *p*<0.05).

**Conclusions:**

Increasing the consumption of B1, B2, and B6 may significantly lower values of indices of obesity and cardiac function regardless of sex differences. Thus reducing the occurrence of obesity and related coronary heart diseases.

## Introduction

Micronutrients work as coenzymes, cocatalysts, and buffers and are essential for various cellular and biochemical processes, such as releasing energy for synthesis, movement, and other processes. ^1^ Due to their impact on intracellular homocysteine concentration and oxidative stress, subclinical deficiencies in several micronutrients have the potential to significantly slow the development of chronic disease. ^2^ When the consumption of a micronutrient falls below the current recommended dietary amount, a significant chronic metabolic disturbance may result from the nutrient deficiencies such as polycystic ovarian syndrome (PCOS), insulin resistance, obesity, and metabolic syndrome. ^3^^–^^5^ In recent years, many studies approved that nutrients could play a vital role in obesity and metabolic syndrome prevention. Also, energy-dense foods with deficit nutrients contribute to the development of obesity, metabolic syndrome, and inflammation. ^4^^,^^6^

B vitamins play a crucial role in the balance and metabolism of energy. ^7^ Energy metabolism mainly benefits from the B-complex vitamins. Specifically, decarboxylation, transamination, acylation, oxidation, and reduction of substrates that are ultimately employed in energy intake require thiamin, riboflavin, niacin, and vitamin B6. One or more of these are also necessary for synthesizing steroids, fatty acids, cholesterol, amino acids, and glucose. ^8^ B-vitamin deficiency may impact energy metabolism, and cause insulin resistance, chronic inflammation, obesity, and elevated oxidative stress, which may contribute to the pathophysiology of metabolic syndrome. ^9^^–^^11^ B-vitamin deficiency has been approved in individuals with obesity and metabolic syndrome. ^2^^,^^12^ Moreover, thiamin (B1) and riboflavin (B2) intake are inversely associated with body mass index (BMI). ^2^^,^^7^ As energy metabolism may be affected by deficiencies in biotin, niacin (B3), folate, and vitamins B6 and B12, which may enhance the generation of reactive oxidants and inflammatory reactions, lipogenesis may also be promoted, which may increase obesity. ^13^^,^^14^ Additionally, B vitamins play a vital role (methyl group donor and act as a coenzyme) in DNA methylation, which is important for body adiposity regulation ^15^ and is associated with BMI. ^16^

Wu *et al.*, ^6^ suggested that a higher intake of vitamins B1, B2, B3, B6, and dietary folate were associated with a reduced risk of metabolic syndrome. Also, numerous cardiovascular diseases (CVDs), and risk factors, such as type 1 and type 2 diabetes, obesity, chronic vascular inflammation, dyslipidemia, heart failure, myocardial infarction, and depression, have been linked to B1, B3, and B6 deficiency. ^12^^,^^17^

Dietary intake of B vitamins has been found to be inversely related to CVD, as their attribution in lowering CVD risk factors, such as plasma homocysteine, serum triglycerides, and C-reaction protein. ^18^ These risk factors have been linked to the atherosclerotic process ^19^ and endothelial dysfunction, ^20^ which engage in CVD development. B vitamins play a role in the metabolism of one carbon unit, therefore each of these nutrients may have an impact on the pathological development of CVD by reducing homocysteine concentrations, methylation processes, and oxidative stress. ^21^^,^^22^

Furthermore, nicotinamide adenine dinucleotide (NAD) and nicotinamide adenine dinucleotide phosphate (NADP) are redox mediator coenzymes that are precursors of B3, ^23^ which has also been demonstrated to enhance levels of high-density lipoprotein cholesterol (HDL), lower levels of triglycerides (TG), very low levels of low-density lipoprotein cholesterol (VLDL-C), and dramatically diminish the risk of CVD and the progression of atherosclerosis. ^24^ Based on the aforementioned, not enough data are available to cover the association between energy-releasing vitamins with indices of obesity and cardiac function. So, this study aimed to assess the impact of energy-releasing B-vitamins intake (B1, B2, B3, and B6) on selected indices of obesity and cardiac function.

## Methods

### Study design, participants, and ethical approval

A cross-sectional study design was used and conducted on 491 apparently healthy randomly selected male and female adults, university employees and students, aged between 18-64 years old (between January and May 2019) at The Hashemite University, Jordan. The participants were asked to sign informed consent before participation; an example of the consent form can be found as *Extended data.* ^25^ Exclusion criteria were pregnant or lactating women, subjects with incomplete anthropometric measurements and/or biochemical data. The Raosoft calculator (online) was used to calculate the sample size based on the Department of Statistics in 2020. Adults represented 52.2% (5,621,970) of Jordan's population (10,806,000). With a 5% error margin, 95% CI, and 50% response distribution, the minimum sample size was 385 participants.

The study was approved by The Hashemite University Ethics Committee, which authorized the protocols, tools, and procedures. All procedures were carried out according to the Declaration of Helsinki and the Institutional Board Review (IRB) (No.7/13/2018/2019; date approved 13/7/2018).

### Data collection

Demographic data, including age, sex, marital status, education level, lifestyle data (such as smoking status and physical activity), medical history, and family history of diseases were gathered through conducting a face-to-face structured interview in a private room using closed-ended questions, which can be found as *Extended data.* ^26^ The interviewer asked the participants questions and then filled out the data form with the participants responses to ensure the accuracy of the filled data (took place over 15-18 minutes). The participants were asked to provide verbal and written consent before participation. Subjects who consumed nutritional supplements like multivitamins or subjects who adhered to restrictive diets such as weight loss diets or have food allergies, pregnant and lactating women were excluded.

### Qualitative variables

Socio-demographic data, including age, sex, marital status, education level, lifestyle data (such as smoking status and physical activity), medical history, and family history of diseases, were gathered through professional staff face-to-face interviews. These data were collected using a designed list of questions created for the study and put through a reliability test, after completion by 25 participants in a pilot study (α=0.77). The first 24 hours of dietary intake were recorded during these interviews. Moreover, participants were asked to schedule another meeting for fasting blood sample testing and collecting the second 24-hour dietary recall.

### Quantitative variables

Each participant had a fasting blood sample drawn after 8-12 hours of fasting, which was subsequently examined using a standardized procedure. A biochemical autoanalyzer (SEAL - AA3 HR - Colorimeter) was used to analyze the triglyceride (TG) and high-density lipoprotein (HDL). Using standardized techniques, well-trained examiners measured anthropometric indices. Participants were measured for body weight and height, wearing light clothing and bare feet, to the nearest 0.1 kg and 0.1 cm, respectively. With the individual standing, the waist circumference was measured using a flexible anthropometric tape on the horizontal plane halfway between the lowest rib and the iliac crest. At the place where the buttocks are at their widest comprehensive hip circumference was measured over thin clothing. The measurements of both circumferences were made to the nearest 0.1 cm. BMI was computed as weight divided by height square (kg)/(m ^2^). The waist-to-height ratio was calculated (waist measurement divided by height measurement). Furthermore, in this study, five obesity indices were considered and calculated using standard formulas as follows: conicity index (CI) = WC (m)/(0.109 × √ (Bodyweight (kg)/Height (m)) ^27^; body adiposity index (BAI) = hip circumference (cm)/height (m)1.5–18) ^28^; abdominal volume index (AVI) = (2 cm (waist) ^2^ + 0.7 cm (waist-hip)2)/1,000) ^27^; body roundness index (BRI0 = 365.2 − 365.5 × √(1 − ((WC/2π) ^2^)/((0.5 × height)) ^2^)) ^29^; and weight-adjusted-waist index (WWI) = WC (cm) divided by the square root of weight (kg). ^30^

Moreover, cardiac function indices were considered and calculated, including an atherogenic index of plasma (AIP), lipid accumulation product (LAP), and cardiometabolic index (CMI), based on the following equations: LAP for males = (waist (cm)−65) × TG concentration (mmol/l), and for female = (waist (cm)−58) × TG concentration (mmol/l); CMI = TG/HDL-C ×(waist-to-height); AIP = Log (TG/HDL-C). ^31^

### Dietary assessment

Two nonconsecutive 24-hour day recalls measured dietary intake (weekday and weekend). The participants were instructed to recollect and list all foods and beverages they had from midnight to midnight the day before, along with their quantity, cooking technique, and the names of brands. To ascertain the typical dietary intake of B1, B2, B3, and B6 vitamins, the database of ingested items was connected to ESHA Food Processor Nutrition Analysis (RRID: SCR_022528) Software (version 11:0; ESHA Research). The average total intake of the selected vitamins after adjustment for energy intake was calculated, then using percentiles (33% and 66%); the subject intake was categorized as tertiles (T1 presents the lower intake, T3 presents medium intake, and T3 presents the higher intake). T1 was a value less than the 33 percentiles (B1: <1.6536; B2: <1.4200; B3: < 25.6736; B6: < 1.3700), T2 was the value between 33 and 66 percentiles (B1: 1.6536-2.2800; B2: 1.4200-1.9400; B3: 25.6736-36.6252; B6: 1.3700-1.9300), and T3 was the value above the 66 percentiles (B1: >2.2800; B2: >1.9400; B3: >36.6252; B6: >1.9300).

### Bias

To prevent sample bias, we selected a representative sample size, which was calculated based on the total Jordanian population using online software Raosoft (Raosoft Inc., free online software, Seattle, WA, USA). Also, the incomplete anthropometric measurements and/or biochemical data were excluded to eliminate information bias. However, there were no self-reporting data to bypass recall bias, the interviewers were trained for data collection.

### Study size

The sample size was calculated using the calculator Raosoft based on the Department of Statistics in 2020, adults represented 52.2% (5,621,970) of the total population (10,806,000) in Jordan. With a 5% error margin, 95% CI, and 50% response distribution, and after addition of 10%, the minimum sample size was 424 participants. The total number of final participants was 491.

### Statistical analysis

Shapiro–Wilk test was used to verify the normality of variables. To describe the sample, descriptive statistical tests were used. Means and standard deviations (means± SD) were used to express continuous variables. Categorical variables, on the other hand, are displayed as frequencies and percentages (n (%)). Age groups stratified this analysis.

Means and standard deviation to present the continuous variables were calculated using an ANOVA test. Based on the sample distribution and test statistics, inferential statistics were applied, including the student’s t-test, chi-squared test, and independent samples t-test. The energy-adjusted intakes of B-vitamins were calculated by adding the expected mean nutrient intake of the study population to the residual derived from the regression analysis. ^32^ This energy adjustment method was computed from the residuals of the regression model with total energy intake as the independent variable and the nutrient as the dependent variable. Pearson correlation coefficient was performed to determine the correlation between obesity indices and macronutrient intake. The percentage change in the index explained by nutrient intake was calculated using r-square from linear regression analysis considering age and sex as confounding factors. The statistical significance was set at *p* < 0.05. IBM SPSS Statistics (RRID: SCR_016479) version 25 (IBM, Chicago, IL, USA) was used.

## Results

### Descriptive data

From the total sample, 198 participants were aged between 20-34 years old, 169 were aged 35-44 years old, and 124 were aged 45-65 years old. Participants aged between 20-34 years old had a mean weight of 74.04 ± 15.72 kg, WC 89.69 ± 13.15 cm, HC 102.98 ± 10.09 cm, and BMI 25.68 ± 4.96 kg/m ^2^. The participants aged between 35-44 years old had a mean weight of 79.48 ± 15.32 kg, WC 97.18 ± 12.65 cm, HC 106.26 ± 9.62 cm, and BMI 28.00 ± 4.58 kg/m ^2^. While participants aged between 45-65 years old had a mean weight of 84.40 ± 15.51 kg, WC 105.18 ± 15.59 cm, HC 108.22 ± 12.02 cm, and BMI 30.21 ± 5.94 kg/m ^2^. Most of the participants in the different age groups were male (66.2, 68.0 and 79.0%, respectively) ( Table 1). ^33^ A total of 51.5% of participants aged 20-34 years old had a school-level education, while 57.4% and 50.8% of participants aged 35-44 and 45-65 years old, respectively, had a university-level education. Most of the participants aged 35-44 and 45-65 years old were single, whereas 43.9% of participants aged 20-34 years old were married and 55.6% were single. Participants aged 20-34 years old were either normal or overweight (49.5% and 30.3%, respectively). Participants aged 35-44 and 45-65 years old were either overweight (40.2% and 42.7%, respectively) or obese (32.0% and 41.1%, respectively). In addition, a larger proportion of the participants of all ages were physically active and smokers. Almost all the participants of different ages were disease-free ( Table 1).

**Table 1.** The general characteristics of the study population (n=491).

Variables	Mean ± SD			*p*-value *
	20-34 y (n = 198)	35-44 y (n = 169)	45-65 y (n = 124)	
Weight (kg)	74.04 ± 15.72	79.48 ± 15.32	84.40 ± 15.51	<0.001 **
Waist circumference (WC) (cm)	89.69 ± 13.15	97.18 ± 12.65	105.18 ± 15.59	<0.001 **
Hip circumference (HC) (cm)	102.98 ± 10.09	106.26 ± 9.62	108.22 ± 12.02	<0.001 **
Body mass index (BMI) (kg/m ^2^)	25.68 ± 4.96	28.00 ± 4.58	30.21 ± 5.94	<0.001 **
	**n (%)**			
**Sex**				
Male	131 (66.2)	115 (68.0)	98 (79.0)	0.038
Female	67 (33.8)	54 (32.0)	26 (21.0)	
**Education level**				
School education level	102 (51.5)	72 (42.6)	61 (49.2)	0.221
University education level	96 (48.5)	97 (57.4)	63 (50.8)	
**Marital status**				
Married	87 (43.9)	148 (87.6)	118 (95.2)	<0.001 **
Single	110 (55.6)	18 (10.7)	4 (3.2)	
Divorced	1 (0.5)	2 (1.2)	1 (0.8)	
Widow	0 (0.0)	1 (0.6)	1 (0.8)	
**Body mass index categories**				
Underweight	8 (4.0)	1 (0.6)	0 (0.0)	<0.001 **
Normal weight	98 (49.5)	46 (27.2)	20 (16.1)	
Overweight	60 (30.3)	68 (40.2)	53 (42.7)	
Obese	32 (16.2)	54 (32.0)	51 (41.1)	
**Physical activity**				
Active	128 (65.6)	90 (53.3)	63 (50.8)	0.022
Inactive	70 (35.4)	79 (46.7)	61 (49.2)	
**Smoking**				
Yes	82 (41.4)	61 (36.1)	57 (46.0)	0.002 **
No	63 (31.8)	67 (39.6)	32 (25.8)	
Ex-smoker	9 (4.5)	15 (8.9)	19 (15.3)	
Passive smoking	44 (22.2)	26 (15.4)	16 (12.9)	
**Having diabetes multiuse**				
Yes	0 (0.0)	6 (3.6)	22 (17.7)	<0.001 **
No	198 (100.0)	163 (96.4)	102 (82.3)	
**Having dyslipidemia**				
Yes	1 (0.5)	11 (6.5)	16 (12.9)	<0.001 **
No	197 (99.5)	158 (93.5)	108 (87.1)	
**Having hypertension**				
Yes	2 (1.0)	7 (4.1)	18 (14.5)	<0.001 **
No	196 (99.0)	162 (95.9)	106 (85.5)	
**Having heart disease**				
Yes	1 (0.5)	3 (1.8)	8 (6.5)	0.003 **
No	197 (99.5)	166 (98.2)	116 (93.5)	

* *p*-value < 0.05 considered statistically significant (2-tailed).

** *p*-value is significant at the 0.01 level (2-tailed).

### Main results

The average total intake of the macronutrients and selected B vitamins were 3,050.09 ± 914.90 Kcal for energy, 364.32 ± 118.76 g for carbohydrate, 98.28 ± 41.29 g for protein, 134.89 ± 58.84 g for fat, 2.09 ± 0.82 mg for unadjusted B1 vitamin, 1.79 ± 0.83 mg for unadjusted B2 vitamin, 32.84 ± 14.16 mg for unadjusted B3 vitamin, and 2.02 ± 6.29 mg for unadjusted B6 vitamin. The mean intake and tertiles range for adjusted B vitamins were: 2.09 ± 0.59 mg (T1: <1.89; T2: 1.89-2.38; T3: >2.38) for energy-adjusted B1 vitamin, 1.79 ± 0.70 mg (T1: <1.54; T2: 1.54-1.88; T3: >1.88) for energy-adjusted B2 vitamin, 32.8 ± 12.34 mg (T1: <26.11; T2: 26.11-36.33; T3: >36.33) for energy-adjusted B3 vitamin, and 2.02 ± 6.22 mg (T1: <1.47; T2: 1.47-2.06; T3: >2.06) for energy-adjusted B6 vitamin.

The mean score of indices of obesity and cardiac function among the adjusted B vitamins tertiles are shown in Table 2. Among adjusted vitamin B1 intake tertiles, the CI and AVI index score significantly decreased as the intake of B1 intake increased (CI: T1 = 1.31 ± 0.01 *vs.* T3 = 1.28 ± 0.01, *p* = 0.031; AVI: T1=20.75 ± 0.64 *vs.* T3 = 18.14 ± 0.43, *p* = 0.02). Among adjusted B6 tertiles the score of the CI (T1 = 1.33 ± 0.01 *vs.* T3 = 1.27 ± 0.01, *p* < 0.001), AVI (T1 = 20.62 ± 0.57 *vs.* T3 = 17.43 ± 0.36, *p* < 0.001), BRI (T1 = 2.19 ± 0.09 *vs.* T3 = 1.60 ± 0.06, *p* < 0.001), WWI (T1 = 11.16 ± 0.09 *vs.* T3 = 10.71 ± 0.06, *p* < 0.001), LAP (T1 = 69.29 ± 4.48 *vs.* T3 = 50.31 ± 3.09, *p* = 0.048), AIP (T1 = 0.55 ± 0.03 *vs.* T3 = 0.46 ± 0.02, *p* = 0.034) decreased significantly as we moved from T1 to T3.

**Table 2.** The mean scores of indices of obesity and cardiac function among the adjusted B-vitamins tertiles.

Nutrient	Mean ± SEM			*p*-value *
	T1 (n = 165)	T2 (n = 163)	T3 (n = 163)	
**Vitamin B1**				
Conicity Index (CI)	1.31 ± 0.01	1.31 ± 0.01	1.28 ± 0.01	0.031 *
Body adiposity index (BAI)	-17.49 ± 0.01	-17.50 ± 0.01	-17.50 ± 0.01	0.582
Abdominal volume index (AVI)	20.75 ± 0.64	19.14 ± 0.39	18.14 ± 0.43	0.002 **
Body Roundness Index (BRI)	2.01 ± 0.11	1.79 ± 0.07	1.72 ± 0.08	0.059
Weight-adjusted-waist index (WWI)	10.90 ± 0.10	10.94 ± 0.07	10.79 ± 0.07	0.275
Cardiometabolic index (CMI)	2.59 ± 0.20	2.44 ± 0.20	2.68 ± 0.28	0.734
Lipid accumulation product (LAP)	61.92 ± 4.78	56.83 ± 3.36	62.47 ± 7.78	0.728
Atherogenic index of plasma (AIP)	0.53 ± 0.03	0.48 ± 0.02	0.48 ± 0.03	0.428
**Vitamin B2**				
Conicity Index (CI)	1.31 ± 0.01	1.29 ± 0.01	1.29 ± 0.01	0.442
Body adiposity index (BAI)	-17.51 ± 0.01	-17.49 ± 0.01	-17.51 ± 0.01	0.259
Abdominal volume index (AVI)	19.51 ± 0.65	18.94 ± 0.37	19.02 ± 0.47	0.693
Body Roundness Index (BRI)	1.82 ± 0.11	1.83 ± 0.06	1.77 ± 0.08	0.816
Weight-adjusted-waist index (WWI)	10.91 ± 0.10	10.89 ± 0.06	10.83 ± 0.07	0.773
Cardiometabolic index (CMI)	2.90 ± 0.48	2.54 ± 0.15	2.34 ± 0.17	0.359
Lipid accumulation product (LAP)	67.85 ± 12.38	59.26 ± 3.21	55.29 ± 3.80	0.400
Atherogenic index of plasma (AIP)	0.48 ± 0.03	0.50 ± 0.02	0.48 ± 0.03	0.832
**Vitamin B3**				
Conicity Index (CI)	1.30 ± 0.01	1.29 ± 0.01	1.30 ± 0.01	0.866
Body adiposity index (BAI)	-17.50 ± 0.01	-17.50 ± 0.01	-17.51 ± 0.01	0.644
Abdominal volume index (AVI)	19.20 ± 0.44	18.92 ± 0.51	19.18 ± 0.44	0.890
Body Roundness Index (BRI)	1.82 ± 0.07	1.80 ± 0.09	1.80 ± 0.07	0.972
Weight-adjusted-waist index (WWI)	10.89 ± 0.06	10.86 ± 0.08	10.87 ± 0.07	0.943
Cardiometabolic index (CMI)	2.33 ± 0.23	2.48 ± 0.17	2.86 ± 0.31	0.292
Lipid accumulation product (LAP)	55.54 ± 4.09	58.57 ± 3.98	65.70 ± 8.41	0.460
Atherogenic index of plasma (AIP)	0.45 ± 0.03	0.50 ± 0.02	0.52 ± 0.03	0.209
**Vitamin B6**				
Conicity Index (CI)	1.33 ± 0.01	1.30 ± 0.01	1.27 ± 0.01	<0.001 **
Body adiposity index (BAI)	-17.49 ± 0.01	-17.50 ± 0.01	-17.51 ± 0.01	0.051
Abdominal volume index (AVI)	20.62 ± 0.57	19.09 ± 0.45	17.43 ± 0.36	<0.001 **
Body Roundness Index (BRI)	2.19 ± 0.09	1.76 ± 0.08	1.60 ± 0.06	<0.001 **
Weight-adjusted-waist index (WWI)	11.16 ± 0.09	10.84 ± 0.07	10.71 ± 0.06	<0.001 **
Cardiometabolic index (CMI)	2.78 ± 0.20	2.79 ± 0.36	2.24 ± 0.15	0.163
Lipid accumulation product (LAP)	69.29 ± 4.48	64.63 ± 9.13	50.31 ± 3.09	0.048 *
Atherogenic index of plasma (AIP)	0.55 ± 0.03	0.48 ± 0.03	0.46 ± 0.02	0.034 *

* *p*-value < 0.05 considered statistically significant (2-tailed).

** *p*-value is significant at the 0.01 level (2-tailed).

The association between indices of obesity and cardiac function with the adjusted energy intake of B vitamins among the total sample and based on sex are illustrated in Table 3. The energy-adjusted intake of B1 for the total sample had a significantly inverse weak correlation with AVI (r = -0.156, *p* = 0.001) and BRI (r = -0.111, *p* = 0.014). Among male participants, similar correlations were detected between energy adjusted B1 intake and BAI (r = -0.207, *p* < 0.001), AVI (r = -0.145, *p* = 0.007), and BRI (r = -0.163, *p* = 0.002). While, among female participants, there was a significant weak inverse correlation between BAI and energy adjusted B2 (r = -0.180, *p* = 0.029) and B6 intake (r = -0.212, *p* = 0.010). Moreover, there was a significant weak inverse association between energy adjusted B6 intake and AVI (r = -0.252, *p* = 0.002), BRI (r = -0.230, *p* = 0.005), and LAP (r = -0.179, *p* = 0.030) indices.

**Table 3.** The correlation between adjusted for energy vitamin B1, B2, B3, and B6 intake with obesity and cardiac function indices.

B-vitamins	Statistical analysis	Indices of obesity and cardiac function							
		CI	BAI	AVI	BRI	WWI	CMI	LAP	AIP
**Total participants**									
B1 intake (mg)	Pearson r	-0.078	-0.064	-0.156	-0.111	-0.041	0.040	0.040	-0.022
	*p*-value	0.084	0.155	0.001 **	0.014 *	0.362	0.372	0.371	0.631
B2 intake (mg)	Pearson r	-0.047	-0.036	-0.058	-0.040	-0.029	-0.054	-0.051	-0.010
	*p-*value	0.298	0.421	0.203	0.379	0.518	0.235	0.257	0.823
B3 intake (mg)	Pearson r	-0.001	-0.031	-0.001	-0.010	-0.007	0.049	0.034	0.071
	p-value	0.989	0.500	0.979	0.829	0.875	0.276	0.450	0.115
B6 intake (mg)	Pearson r	-0.064	-0.014	-0.051	-0.055	-0.070	-0.022	-0.028	-0.013
	p-value	0.156	0.756	0.263	0.222	0.120	0.620	0.530	0.772
**Male participants**									
B1 intake (mg)	Pearson r	-0.020	-0.207	-0.145	-0.163	-0.040	0.078	0.079	0.030
	p-value	0.716	<0.001 **	0.007 **	0.002 **	0.465	0.149	0.141	0.583
B2 intake (mg)	Pearson r	-0.038	0.010	-0.014	-0.006	-0.028	-0.019	-0.026	0.037
	p-value	0.488	0.846	0.799	0.916	0.604	0.725	0.629	0.493
B3 intake (mg)	Pearson r	-0.020	-0.042	-0.025	-0.036	-0.027	0.095	0.058	0.093
	p-value	0.706	0.441	0.650	0.502	0.619	0.077	0.281	0.085
B6 intake (mg)	Pearson r	-0.070	-0.015	-0.049	-0.058	-0.076	-0.020	-0.025	-0.007
	p-value	0.194	0.786	0.369	0.280	0.157	0.710	0.650	0.890
**Female participants**									
B1 intake (mg)	Pearson r	0.137	-0.127	0.042	0.048	0.147	0.073	0.080	0.105
	*p*-value	0.097	0.126	0.618	0.565	0.076	0.381	0.336	0.206
B2 intake (mg)	Pearson r	0.039	-0.180	-0.079	-0.082	0.022	-0.098	-0.093	-0.033
	p-value	0.639	0.029 *	0.339	0.322	0.788	0.239	0.263	0.690
B3 intake (mg)	Pearson r	0.039	-0.015	0.046	0.040	0.036	-0.077	-0.065	0.021
	p-value	0.642	0.854	0.584	0.634	0.663	0.357	0.435	0.800
B6 intake (mg)	Pearson r	-0.136	-0.212	-0.252	-0.230	-0.131	-0.111	-0.179	-0.112
	p-value	0.100	0.010 **	0.002 **	0.005 **	0.112	0.179	0.030 *	0.175

CI: Conicity Index; BAI: Body adiposity index; AVI: Abdominal volume index; BRI: Body Roundness Index; WWI: Weight-adjusted-waist index; CMI: Cardiometabolic index; LAP: Lipid accumulation product; AIP: Atherogenic index of plasma.

** Correlation is significant at the 0.01 level (2-tailed).

* Correlation is significant at the 0.05 level (2-tailed).

For more details, the percentage changes in indices of obesity and cardiac function explained by energy-adjusted B vitamins intakes using the linear regression model are presented in Table 4. Among B vitamins, only vitamin B1 significantly explained 2.43% of changes observed in the AVI score and 1.24% of changes observed in the BRI score ( *p* < 0.05).

**Table 4.** Changes in obesity and cardiac function indices based on B vitamin intake using the linear regression model.

Model	R	R Square change	% change	*p-*value for change *
**Conicity Index (CI)**				
Adjusted Vitamin B1	0.078	0.006	0.61%	0.084
Adjusted Vitamin B2	0.047	0.002	0.22%	0.298
Adjusted Vitamin B3	0.001	0.000	0.00%	0.989
Adjusted Vitamin B6	0.064	0.004	0.41%	0.156
**Body adiposity index (BAI)**				
Adjusted Vitamin B1	0.064	0.004	0.41%	0.155
Adjusted Vitamin B2	0.036	0.001	0.13%	0.421
Adjusted Vitamin B3	0.031	0.001	0.09%	0.500
Adjusted Vitamin B6	0.014	0.000	0.02%	0.756
**Abdominal volume index (AVI)**				
Adjusted Vitamin B1	0.156	0.024	2.43%	0.001 *
Adjusted Vitamin B2	0.058	0.003	0.33%	0.203
Adjusted Vitamin B3	0.001	0.000	0.00%	0.979
Adjusted Vitamin B6	0.051	0.003	0.26%	0.263
**Body Roundness Index (BRI)**				
Adjusted Vitamin B1	0.111	0.012	1.24%	0.014 *
Adjusted Vitamin B2	0.040	0.002	0.16%	0.379
Adjusted Vitamin B3	0.010	0.000	0.01%	0.829
Adjusted Vitamin B6	0.055	0.003	0.31%	0.222
**Weight-adjusted-waist index (WWI)**				
Adjusted Vitamin B1	0.041	0.002	0.17%	0.362
Adjusted Vitamin B2	0.029	0.001	0.09%	0.518
Adjusted Vitamin B3	0.007	0.000	0.01%	0.875
Adjusted Vitamin B6	0.070	0.005	0.49%	0.120
**Cardiometabolic index (CMI)**				
Adjusted Vitamin B1	0.040	0.002	0.16%	0.372
Adjusted Vitamin B2	0.054	0.003	0.29%	0.235
Adjusted Vitamin B3	0.049	0.002	0.24%	0.276
Adjusted Vitamin B6	0.022	0.001	0.05%	0.620
**Lipid accumulation product (LAP)**				
Adjusted Vitamin B1	0.040	0.002	0.16%	0.371
Adjusted Vitamin B2	0.051	0.003	0.26%	0.257
Adjusted Vitamin B3	0.034	0.001	0.12%	0.450
Adjusted Vitamin B6	0.028	0.001	0.08%	0.530
**Atherogenic index of plasma (AIP)**				
Adjusted Vitamin B1	0.022	0.000	0.05%	0.631
Adjusted Vitamin B2	0.010	0.000	0.01%	0.823
Adjusted Vitamin B3	0.071	0.005	0.51%	0.115
Adjusted Vitamin B6	0.013	0.000	0.02%	0.772

* *p*-value < 0.05 considered statistically significant (2-tailed).

## Discussion

Obesity is considered to be one of the malnutrition forms, which develops individually and as a result of unbalanced nutrition. ^2^ Based on The World Health Organization (WHO), obesity is defined as the “double burden of malnutrition” and indicates that it may occur more frequently in socioeconomically underdeveloped and poorly nourished societies side by side with malnutrition, and in consequence, chronic diseases may develop. ^34^ Vitamins B1 (thiamine), B2 (Riboflavin), B3 (niacin), and B6 (pyridoxine), all of which are vital for the metabolism of energy, carbohydrate, lipid, and amino acid, as well as the regulation of the cellular redox state. ^12^ Some studies have revealed that obesity is associated with low levels of fruit intake, total diet quality, and micronutrient intake compared to normal BMI, which means that people living with obesity may consume fewer micronutrients due to the foods they eat and subsequently may lead to low levels of water-soluble vitamins. ^12^^,^^35^ Also, it has been found that the requirements of B vitamins has been increased for people with obesity, diabetes, and peripheral neuropathy. ^36^^,^^37^

The current study studied the association between dietary intake of B vitamins; specifically, B1, B2, B3, and B6, with indices of obesity and cardiac function were studied. Among these B vitamins, B1 and B6 had a significant association with some of the obesity indices; B2 with BAI indices, and B6 had a significant association with LAP (one of the cardiac function indices) The result revealed that the score of obesity indices CI and AVI decreased with an increase in the dietary intake of vitamin B1. Moreover, the energy-adjusted intake of B1 had a significantly inverse weak correlation with AVI and BRI among the total sample and male participants. Energy-adjusted B1 vitamin significantly explained 2.43% of changes observed in the AVI and 1.24% of changes observed in the BRI indices ( *p* < 0.05). In addition, based on this study result, increasing the intake of B6 led to reductions in the score of obesity (CI, AVI, BRI, WWI) and cardiac function indices (LAP and AIP).

**Figure 1.**
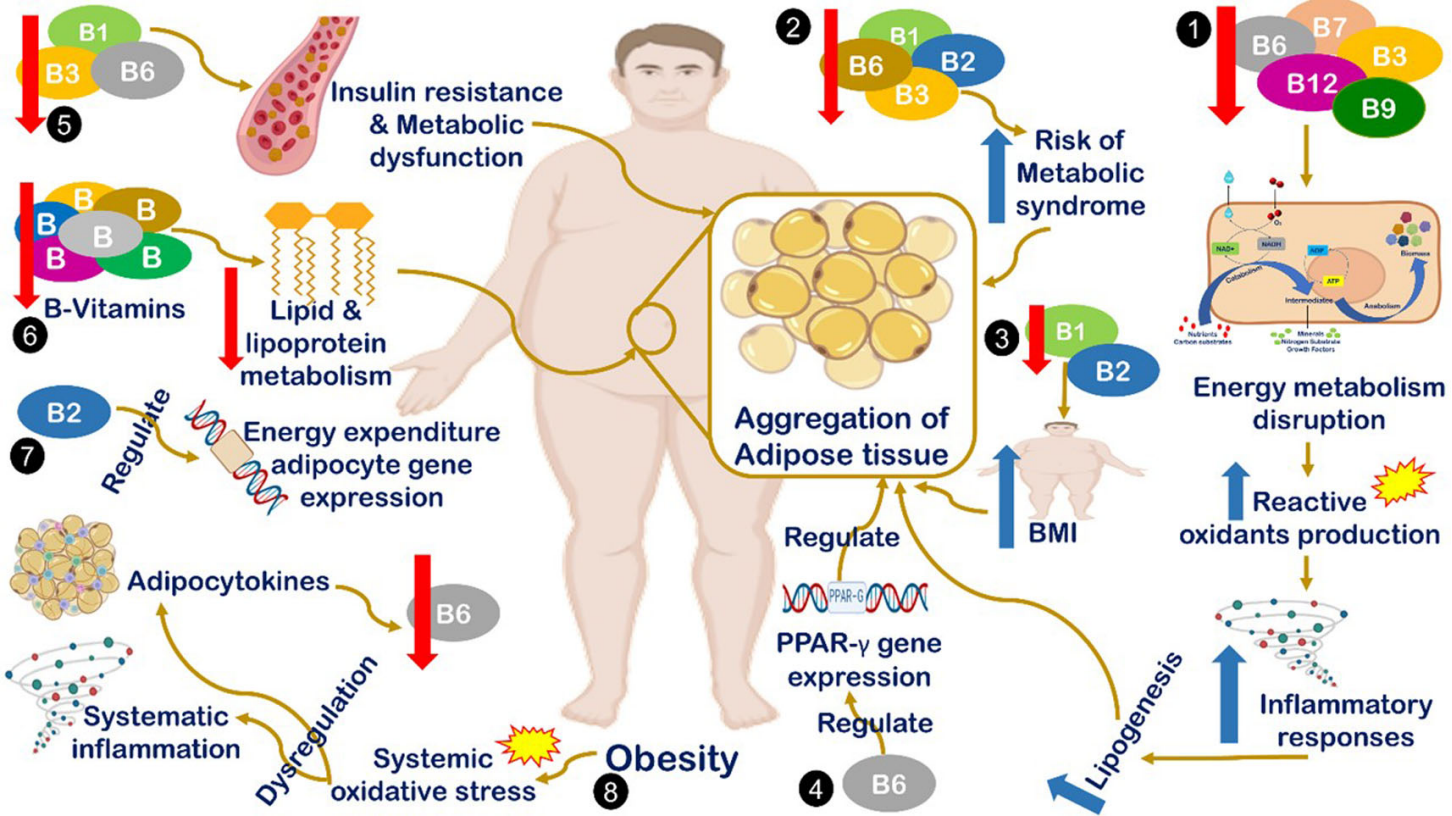
Mechanism of interaction between reduction level of serum B-complex and obesity. (1) Deficiencies in B7, B6, B9, and B12 lead to energy metabolism disruption, which induces the production of reactive oxygen species, inflammation, and lipogenesis. ^7^ (2) Reduced levels of B1, B2, B3, and B6 levels are associated with an increased risk of metabolic syndrome, which is considered one of the major cases of obesity. ^6^ (3) Decreased levels of B1 and B2 are correlated with increased body mass index (BMI). ^7^ (4) B6 regulates the expression of peroxisome proliferator-activated receptor gamma)PPARg(target genes, which play a key role in adipocyte gene expression and adipogenesis. ^45^ (5) Decreased B1, B3, and B6 concentrations are significantly correlated with increased risk of insulin resistance, metabolic dysfunction and obesity. ^46^ (6) B vitamins are fundamental in the lipid and lipoprotein metabolism cascade. Therefore, quantitative or qualitative defects in B vitamins will negatively impact the metabolism of lipid derivatives. ^41^ (7) B2 plays a vital role in energy-expenditure adipocyte gene regulation at the epigenetic level. ^47^ (8) Increased body weight leads to the reduction of vitamin B6 levels by inducing systemic oxidative stress, which leads to stimulating cytokines dysregulation. ^7^^,^^42^

By these results, Kerns *et al.*, reported that 15.5–29% of patients with obesity who seek bariatric surgery have malnutrition disease, specifically, B1 deficiency. ^38^ In line with the present findings, B1 deficiency was found in children with obesity, and most of those cases were subclinical. ^39^ Kardas *et al.*, observed significantly decreased levels of vitamin B2, vitamin B12, and B9 in the obesity and metabolic syndrome groups. ^2^ By contrast, they observed an increased level of B1 with a positive correlation with LDL-C and a negative correlation with HOMA-IR and body weight in the target group. ^2^ Studies show that vitamin B2 deficiency causes functional alterations in adipocytes, and thus has pro-inflammatory and proinsulin effects, resulting from increased adipocytes. ^40^ Moreover, in the subjects with obesity, an inverse correlation was found between vitamin B6 and body weight. ^2^ Gunanti *et al.* (2014) examined the associations between serum B vitamins and vitamin intake with body fat in Mexican American children aged 8–15 years. B1 and B2 intakes were inversely associated with BMI and trunk fat mass (b:21.26, *p* < 0.05; b: 21.37, *p* < 0.05). ^7^ Different suggested mechanisms have been introduced to describe the correlation between B-vitamin deficiency and obesity occurrence, all are summarized in Figure 1. The interaction mechanism between the reduced serum B-complex level and obesity includes the reduction in B1, B2, B3, and B6 levels associated with an increased risk of metabolic syndrome, which is considered one of the major cases of obesity. ^6^ Also, decreases in the levels of B1 and B2 are correlated with increased BMI, ^7^ and B6 regulates the expression of peroxisome proliferator-activated receptor gamma (PPARγ), which is a target gene that plays a key role in adipocyte gene expression and adipogenesis. ^14^ Additionally, decreased B1, B3, and B6 concentrations correlate significantly with an increased risk of insulin resistance, metabolic dysfunction, and obesity. ^41^ Moreover, generally, B vitamins have a fundamental role in the lipid and lipoprotein metabolism cascade. Therefore, quantitative or qualitative defects in B vitamins will negatively impact the metabolism of lipid derivatives. ^42^ Also, B2 plays a vital role in energy-expenditure adipocyte gene regulation at the epigenetic level. ^43^ The final suggested mechanism is that increased body weight leads to the reduction of vitamin B6 levels by inducing systemic oxidative stress, which leads to stimulating adipocytokines dysregulation. ^7^^,^^24^

**Figure 2.**
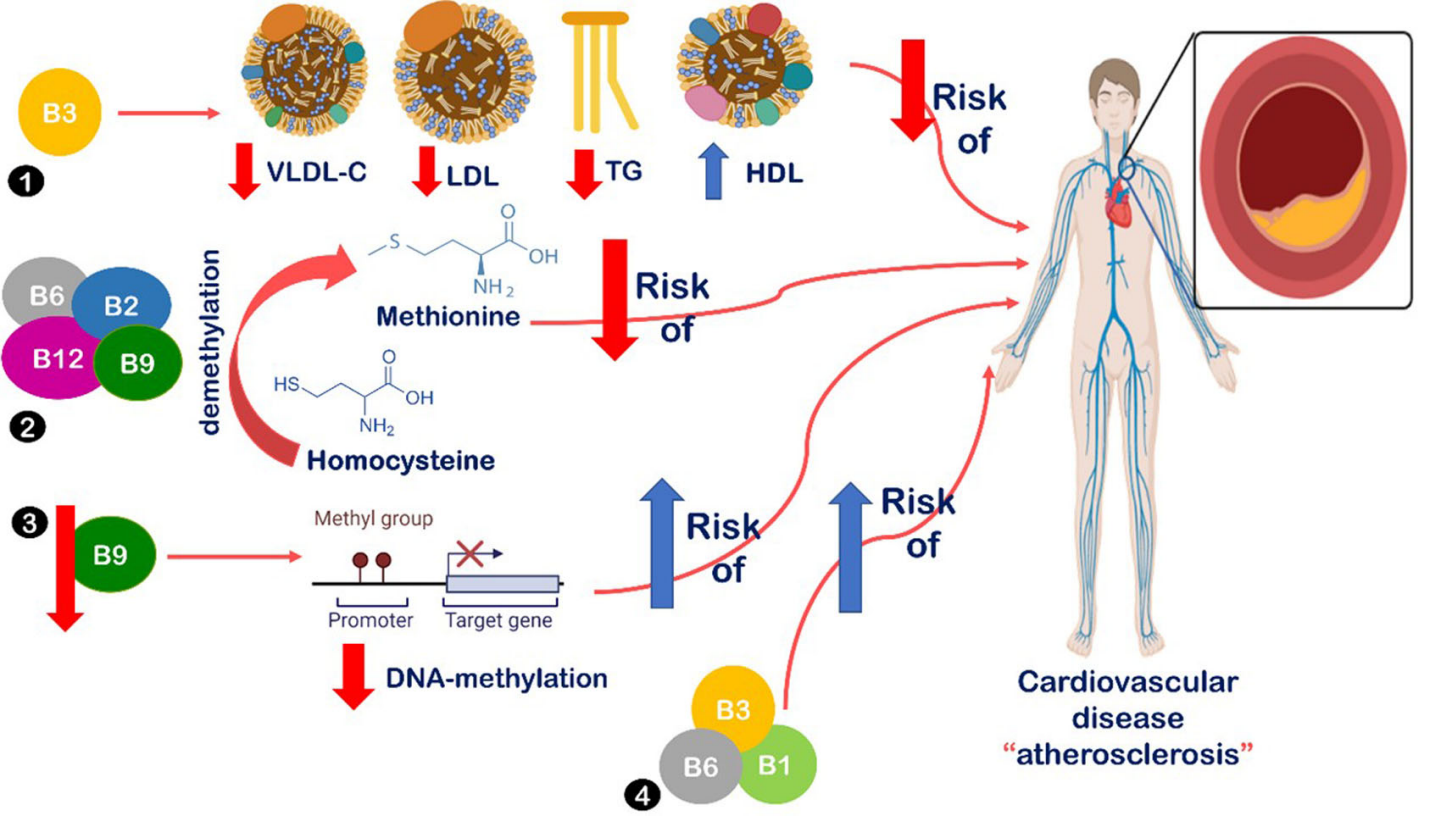
Relationship between B-vitamins intake and cardiovascular disease. (1) B3 intake leads to decreased serum levels of low-density lipoprotein cholesterol (LDL), very low-density lipoprotein cholesterol (VLDL), and triglycerides (TG). Also, increased levels of high-density lipoprotein cholesterol (HDL). ^21^ (2) Vitamins B2, B6, B9, and B12 are vital in homocysteine demethylation into methionine, leading to decreased risk of atherosclerosis. ^15^^,^^21^ (3) Decreased levels of folate intake lead to decreased DNA-methylation, which has been detected in atherosclerotic lesions. ^48^ Also, B6 is associated with reduced risk of metabolic syndrome. ^6^ (4) Low B1, B3, and B6 levels were associated with increased insulin resistance, metabolic dysfunction, and increased risk of atherosclerosis, obesity, metabolic syndrome, diabetes, and cardiovascular disease. ^12^^,^^44^

Studies investigating the prevalence of B1 deficiency among patients with cardiovascular diseases (CVDs) concluded that B1 deficiency could have a role in the development of CVDs. ^17^ Odum *et al.* (2012) found that the mean plasma vitamins B1, B3, B6 and C concentrations of subjects with metabolic syndrome were significantly lower than that of controls ( *p* = 0.001, 0.05, 0.045, 0.001, respectively). Also, 14% and 32% of subjects had inadequate vitamin B1 and vitamin C status, respectively. ^12^ Additionally, Wu *et al.*, found a linear inverse relationship between dietary vitamin B1, niacin, B6, and metabolic syndrome, and a non-linear inverse relationship was found between dietary vitamin B2 and metabolic syndrome. ^6^ It was noted that vitamin B6 supplementation is vital in preventing metabolic syndrome and pyridoxine levels were lower in patients with metabolic syndrome compared to those in healthy patients. ^43^ They suggested that a higher intake of vitamin B1, B2, niacin, B6, and dietary folate equivalent was associated with a reduced risk of metabolic syndrome. ^6^ Not like other results, Kardas *et al.*, found that B1 levels were high in subjects with obesity and metabolic syndrome; they explained this difference by the fact that these individuals typically consumed large quantities of bread and cereals, which are considered major sources of B1. ^2^ Many suggested mechanisms have been introduced to describe the association between B-vitamin deficiency and metabolic syndrome and coronary heart diseases, all are summarized in Figure 2.

B3 intake is suggested to decrease serum levels of LDL, VLDL, and TG, as well as, increase levels of HDL. ^21^^,^^24^ Also, vitamins B2 and B6 have a vital role in homocysteine demethylation into methionine, which leads to decreased risk of atherosclerosis. ^15^^,^^21^ Additionally, B6 is associated with a reduced risk of metabolic syndrome. ^6^ Lastly, low levels of B1, B3, and B6 are associated with increased insulin resistance, metabolic dysfunction, and increased risk of atherosclerosis, obesity, metabolic syndrome, diabetes, and cardiovascular disease. ^12^^,^^44^

This study is one of the early studies that placed importance on examining the relationship between inadequate dietary intake of energy-releasing B vitamins, and each of the indices of obesity and cardiac function in healthy individuals. There are not enough studies that clarify the correlation between B vitamins and indices of obesity and cardiac function. This provides the scope to highlight the significance of dietary consumption and the part that nutritional inadequacies play in the prevalence of obesity and heart disease.

The present study had certain limitations. First, because the study was cross-sectional, it was impossible to conclusively establish a causal link between obesity, cardiac function indices, and dietary intake of B vitamins. Second, data were collected from one place, which affects the generalizability of conclusions that cannot reflect the society’s situation. The availability of both conventional and innovative anthropometric measures as well as cardiac function indices is one of our study’s key strengths. Additionally, because the current data were gathered before the COVID-19 pandemic, they can be used to compare studies done before and after the pandemic by researchers in the same field. Another key benefit of the study is the originality of its investigation of the relationship between indicators of cardiac function and several chosen nutrients. We selected a representative sample size to prevent sample bias, which was calculated based on the total Jordanian population using online software. Also, incomplete anthropometric measurements and/or biochemical data have been excluded to eliminate information bias. However, there were no self-reporting data to bypass recall bias, the interviewers were trained for data collection.

## Conclusions

In conclusion, after energy adjustment, obesity indices are inversely associated with B1, B2, and B6 vitamins. Specifically, AVI and BRI were inversely associated with B1 and B6 vitamins, whereas BAI was inversely associated with B2 vitamins among female participants. Only the B6 vitamin was inversely associated with LAP and AIP among cardiac function indices. Increasing the intake of these vitamins (B1, B2, and B6) results in reducing the score of obesity (CI, AVI, BRI, WWI) and cardiac function indices (LAP and AIP), which in consequence may help reduce the occurrence of obesity and related coronary diseases.

## Data Availability

Zenodo: The impact of energy releasing B-vitamin intake on indices of obesity and cardiac function: a cross-sectional study, https://doi.org/10.5281/zenodo.8159117. ^33^ The project contains the following underlying data:
• Supplementary File 1-Energy releasing B vitamins 13-1-2023.sav (Anonymized participant data used in this study) Zenodo: The impact of energy releasing B-vitamin intake on indices of obesity and cardiac function: a cross-sectional study, https://doi.org/10.5281/zenodo.8167423. ^26^ This project contains the list of interview questions used with 24-hour recall form. Zenodo: The impact of energy releasing B-vitamin intake on indices of obesity and cardiac function: a cross-sectional study, https://doi.org/10.5281/zenodo.8167442. ^25^ This project contains an example of the consent form used. Data are available under the terms of the Creative Commons Attribution 4.0 International license (CC-BY 4.0).
