# Supplementary material for: The impact of energy releasing B-vitamin intake on indices of obesity and cardiac function: a cross-sectional study
Source: F1000Res. 2024 Aug 6;12:1382. Originally published 2023 Oct 20. [Version 3] doi: 10.12688/f1000research.139672.3 (PMC11319906; doi:10.12688/f1000research.139672.3)
Supplement: Peer review history

# Reviewer report: Reviewer response for version 3

**DOI:** 10.5256/f1000research.169917.r311209

**Published:** 2024-08-12

**Authors:** Mehran Rahimlou

**Affiliations:** 1 Nutrition, Zanjan University of Medical Sciences, Zanjan, Iran

The authors have been able to make the necessary changes in the manuscript and the manuscript meets the necessary conditions for acceptance.

Is the work clearly and accurately presented and does it cite the current literature?

Yes

If applicable, is the statistical analysis and its interpretation appropriate?

Partly

Are all the source data underlying the results available to ensure full reproducibility?

Yes

Is the study design appropriate and is the work technically sound?

Partly

Are the conclusions drawn adequately supported by the results?

Yes

Are sufficient details of methods and analysis provided to allow replication by others?

Partly

Reviewer Expertise:

Nutrition and diet

I confirm that I have read this submission and believe that I have an appropriate level of expertise to confirm that it is of an acceptable scientific standard.

# Reviewer report: Reviewer response for version 2

**DOI:** 10.5256/f1000research.162623.r304462

**Published:** 2024-08-01

**Authors:** Mehran Rahimlou

**Affiliations:** 1 Nutrition, Zanjan University of Medical Sciences, Zanjan, Iran

This study entitled: The impact of energy releasing B-vitamin intake on indices of obesity and cardiac function: a cross-sectional study is interesting. However, I have some comments.

1. In the introduction section, it would have been better for the researchers to point out why obese people with metabolic syndrome are at risk of deficiency of group B vitamins. 
2. B-vitamin deficiency may impact energy metabolism, and cause insulin resistance, chronic inflammation, obesity, and elevated oxidative stress, which may contribute to the pathophysiology of metabolic syndrome (add more references such as these articles to this sentence: Ghanavati M, et al., 2020 [Ref-1])
3. "Study design, participants, and ethical approval" It is better to explain information about the type of sampling in this section.
4. On the other hand, one of the drawbacks of this study is that all people were selected from a university group, and this significantly reduces the ability to generalize the results to the society, because in sampling based on an institution or organization, the factors There are many confounders that have low generalizability compared to population studies.
5. Researchers should also explain how to calculate the sample size.
6. "Data collection" what is your approach about subjects who consumed nutritional supplements like multi vitamins or subjects who adherent from restrictive diets such as weight loss diets.
7. "The first 24 hours of dietary intake were recorded during these interviews" It would have been better if three days dietary recall (including two working days and one off day) were collected.

Is the work clearly and accurately presented and does it cite the current literature?

Yes

If applicable, is the statistical analysis and its interpretation appropriate?

Partly

Are all the source data underlying the results available to ensure full reproducibility?

Yes

Is the study design appropriate and is the work technically sound?

Partly

Are the conclusions drawn adequately supported by the results?

Yes

Are sufficient details of methods and analysis provided to allow replication by others?

Partly

Reviewer Expertise:

Nutrition and diet

I confirm that I have read this submission and believe that I have an appropriate level of expertise to confirm that it is of an acceptable scientific standard, however I have significant reservations, as outlined above.

## References

[1] : Pistachios and cardiometabolic risk factors: A systematic review and meta-analysis of randomized controlled clinical trials. *Complement Ther Med* .2020;52: 10.1016/j.ctim.2020.102513 102513 10.1016/j.ctim.2020.102513 32951758

# Response

**Published:** 2024-08-01

**Authors:** Buthaina Alkhatib

This study entitled: The impact of energy-releasing B-vitamin intake on indices of obesity and cardiac function: a cross-sectional study is interesting. However, I have some comments.

 1. In the introduction section, it would have been better for the researchers to point out why obese people with metabolic syndrome are at risk of deficiency of group B vitamins. 

 Response: The core of the study hypothesis was to study the effect of a lower dietary intake of B vitamins, which leads to obesity and metabolic syndrome, rather than approving that obese and metabolic syndrome people are at risk of B vitamin deficiency.

 2. B-vitamin deficiency may impact energy metabolism, and cause insulin resistance, chronic inflammation, obesity, and elevated oxidative stress, which may contribute to the pathophysiology of metabolic syndrome (add more references such as these articles to this sentence: Ghanavati M, et al., 2020 [Ref-1])

 Response: Done, Thank you.

 3. "Study design, participants, and ethical approval" It is better to explain information about the type of sampling in this section.

 Response: A random sample.

 4. On the other hand, one of the drawbacks of this study is that all people were selected from a university group, and this significantly reduces the ability to generalise the results to the society, because in sampling based on an institution or organisation, the factors There are many confounders that have low generalisability compared to population studies.

 Response: It has been added to the study limitations. 

 5.  Researchers should also explain how to calculate the sample size.

 Response: Done (The Raosoft calculator (online) was used to calculate the sample size based on the Department of Statistics in 2020. Adults represented 52.2% (5,621,970) of Jordan's population (10,806,000). With a 5% error margin, 95% CI, and 50% response distribution, the minimum sample size was 385 participants.)

 6. "Data collection:" What is your approach to subjects who consumed nutritional supplements like multivitamins or subjects who adhered to restrictive diets such as weight loss diets?

 Response: Yes, we asked about medications, dietary supplements intake, food allergies, and adhesion to a weight reduction diet, and they were excluded (We added sentence about this)

 7. "The first 24 hours of dietary intake were recorded during these interviews." It would have been better if three days of dietary recall (including two working days and one off day) were collected.

 Response: Thank you, there many references whom used 2 days 24 hour recalls for the big samples, rather than using 3 days (sure it is better)

# Reviewer report: Reviewer response for version 2

**DOI:** 10.5256/f1000research.162623.r249706

**Published:** 2024-02-26

**Authors:** Małgorzata Szczuko

**Affiliations:** 1 Department of Biochemistry and Human Nutrition, Pomeranian Medical University, Szczecin, Poland

Position 24 and 25 in the references are identical.

 I have no further comments.

Is the work clearly and accurately presented and does it cite the current literature?

Yes

If applicable, is the statistical analysis and its interpretation appropriate?

Yes

Are all the source data underlying the results available to ensure full reproducibility?

Yes

Is the study design appropriate and is the work technically sound?

Yes

Are the conclusions drawn adequately supported by the results?

Yes

Are sufficient details of methods and analysis provided to allow replication by others?

Yes

Reviewer Expertise:

nutrition, metabolic syndrom, vitamin B

I confirm that I have read this submission and believe that I have an appropriate level of expertise to confirm that it is of an acceptable scientific standard.

# Reviewer report: Reviewer response for version 1

**DOI:** 10.5256/f1000research.152969.r239615

**Published:** 2024-02-12

**Authors:** Małgorzata Szczuko

**Affiliations:** 1 Department of Biochemistry and Human Nutrition, Pomeranian Medical University, Szczecin, Poland

1. The article is clearly written and easy to read. Although it seems that at the same time it does not exhaust the topic in some respects

 2. A major advantage of the study is the number of patients examined and the well-described statistics of the results

 3. the summary lacks a specific summary and conclusion

 4. in the introduction, please also include women with PCOS, in the course of which insulin resistance, metabolic syndrome and obesity develop

 ref [1]

 5. the age of the examined patients is missing in table 1

 6. the following issues should be discussed in the discussion:

 - increased demand for B vitamins in people with obesity and diabetes re [2].

 - peripheral neuropathy ref [3]

 - effects of supplementation and over supplementation of B vitamins

 ref [4]

 7. It is recommended to use the provided literature to improve and improve the article.

Is the work clearly and accurately presented and does it cite the current literature?

Yes

If applicable, is the statistical analysis and its interpretation appropriate?

Yes

Are all the source data underlying the results available to ensure full reproducibility?

Yes

Is the study design appropriate and is the work technically sound?

Yes

Are the conclusions drawn adequately supported by the results?

Yes

Are sufficient details of methods and analysis provided to allow replication by others?

Yes

Reviewer Expertise:

nutrition, metabolic syndrom, vitamin B

I confirm that I have read this submission and believe that I have an appropriate level of expertise to confirm that it is of an acceptable scientific standard, however I have significant reservations, as outlined above.

## References

[1] : The implications of vitamin content in the plasma in reference to the parameters of carbohydrate metabolism and hormone and lipid profiles in PCOS. *J Steroid Biochem Mol Biol* .2020;198: 10.1016/j.jsbmb.2019.105570 105570 10.1016/j.jsbmb.2019.105570 31883924

[2] : Vitamin B status in patients with type 2 diabetes mellitus with and without incipient nephropathy. *Diabetes Res Clin Pract* .2015;107(1) : 10.1016/j.diabres.2014.09.058 157-65 10.1016/j.diabres.2014.09.058 25458341

[3] : Association between neuropathy and B-vitamins: A systematic review and meta-analysis. *Eur J Neurol* .2021;28(6) : 10.1111/ene.14786 2054-2064 10.1111/ene.14786 33619867

[4] : Micronutrient Supplementation to Reduce Cardiovascular Risk. *J Am Coll Cardiol* .2022;80(24) : 10.1016/j.jacc.2022.09.048 2269-2285 10.1016/j.jacc.2022.09.048 36480969

# Response

**Published:** 2024-02-12

**Authors:** Buthaina Alkhatib

1. The article is clearly written and easy to read. Although it seems that at the same time it does not exhaust the topic in some respects
2. **Response: Thank you**
  2. A major advantage of the study is the number of patients examined and the well-described statistics of the results
**Response: thank you**
  3. the summary lacks a specific summary and conclusion
**Response: it has been revised (conclusion: Increasing the consumption of B1, B2, and B6 may significantly lower values of indices of obesity and cardiac function regardless of sex differences. Thus reducing the occurrence of obesity and related coronary heart diseases**
  4. in the introduction, please also include women with PCOS, in the course of which insulin resistance, metabolic syndrome and obesity develop
  ref [1]
**Response: it has been added **
  5. the age of the examined patients is missing in table 1
**Response: the table was prepared regarding the age group, as shown in raw 1)**
  6. the following issues should be discussed in the discussion:
  - increased demand for B vitamins in people with obesity and diabetes re [2].
  - peripheral neuropathy ref [3]
  - effects of supplementation and over-supplementation of B vitamins
  ref [4]
**Response: it has been provided (references 35, 36). But reference no 4 is not within the scope of discussion. **
  7. It is recommended to use the provided literature to improve and improve the article.
**Response: many thanks for your suggestions, it has been provided **
3. **Is the work clearly and accurately presented and does it cite the current literature?**
4. Yes
5. **Is the study design appropriate and is the work technically sound?**
6. Yes
7. **Are sufficient details of methods and analysis provided to allow replication by others?**
8. Yes
9. **If applicable, is the statistical analysis and its interpretation appropriate?**
10. Yes
11. **Are all the source data underlying the results available to ensure full reproducibility?**
12. Yes
13. **Are the conclusions drawn adequately supported by the results?**
14. Yes
